# Intraguild Prey Served as Alternative Prey for Intraguild Predators in a Reciprocal Predator Guild between *Neoseiulus barkeri* and *Scolothrips takahashii*

**DOI:** 10.3390/insects14060561

**Published:** 2023-06-16

**Authors:** Mingxiu Liu, Mian Wang, Yuzhen Nima, Xiaotian Feng, Guangyun Li, Yi Yang, Yaying Li, Huai Liu

**Affiliations:** 1 Key Laboratory of Entomology and Pest Control Engineering, College of Plant Protection, Southwest University, Chongqing 400716, China; lmx0044@126.com (M.L.); w000916m@163.com (M.W.); fxt971221@126.com (X.F.); liguangyun@swu.edu.cn (G.L.); yy18889618711@163.com (Y.Y.); 2 Institute of Vegetable, Tibet Academy of Agriculture and Animal Husbandry Sciences, Lhasa 850032, China; nmayuzhen@126.com

**Keywords:** intraguild predation, predatory mites, predatory thrips, survival, reproduction, prey preference

## Abstract

**Simple Summary:**

Intraguild predation between the introduced enemy *Neoseiulus barkeri* (Hughes) and the native enemy *Scolothrips takahashii* (Priesner) frequently occurs when target prey is scarce. It is hypothesized that intraguild predation is an adaptative strategy for predators, ensuring the persistence of the intraguild predators during periods of prey scarcity. Therefore, we undertook a study to evaluate the effects of the intraguild prey on the survival, development and reproduction of intraguild predators in a reciprocal predator guild with *N. barkeri* and *S. takahashii*. Our results support the hypothesis, showing that intraguild prey enhanced the survival, development and oviposition of intraguild predators, further confirming the effectiveness of intraguild predation as an adaptative strategy.

**Abstract:**

The predatory mites *Neoseiulus barkeri* (Hughes) and the predatory thrips *Scolothrips takahashii* (Priesner) are known as potential biocontrol agents for the two-spotted spider mite *Tetranychus urticae* (Koch). These two predator species occur simultaneously on crops in agricultural ecosystems and are proved to be involved in life-stage specific intraguild predation. The intraguild prey may play a role in securing the persistence of the intraguild predators during food shortage periods. To understand the potential of intraguild prey as food source for intraguild predators in the *N. barkeri* and *S. takahashii* guild at low *T. urticae* densities, the survival, development and reproduction of both predators was determined when fed on heterospecific predators. The choice tests were conducted to determine the preference of the intraguild predator between the intraguild prey and the shared prey. Results showed that 53.3% *N. barkeri* and 60% *S. takahashii* juveniles successfully developed when fed on heterospecific predators. Female intraguild predators of both species fed on intraguild prey survived and laid eggs throughout the experiment. In the choice test, both intraguild predator species preferred their extraguild prey *T. urticae*. This study suggested that intraguild prey served as an alternative prey for intraguild predators prolonged survival and ensured the reproduction of intraguild predators during food shortage, ultimately decreasing the need for the continual release of the predators.

## 1. Introduction

Pests in natural systems are always faced with multiple predators [1,2]. These predators, competing as they do for shared prey, engage in complex interactions such as intraguild predation, which is when predators kill or eat their potential competitors, and is ubiquitous among arthropods [3,4,5,6]. Such interactions between native and introduced biological agents sharing the same prey have been frequently documented [7,8,9]. Releasing natural enemies at low pest densities can lead to food deprivation for predators, and potentially intense intraguild predation of native and introduced natural enemies, which may ultimately lead to exclusion or coexistence of the involved species [10,11,12,13]. For example, releases of *Harmonia axyridis* (Pallas) to control aphids in the field have negatively affected the population of many indigenous natural enemies through intraguild predation [7]. Conversely, intraguild predation can be an adaptative strategy during periods of prey scarcity [14], as intraguild prey can provide nutritional and energetic benefits that increase the survival, development and reproduction of intraguild predators [15]. Moreover, removing intraguild prey from the diet of intraguild predators may not have any negative effects on the fitness of the predators. Thus, feeding on heterospecific predators (intraguild prey) can ensure the persistence of predator populations at low levels of the target prey, decreasing the need for the continual release of the predators [16].

Numerous studies have reported that intraguild prey could be utilized as a food source for intraguild predators, even if the quality of intraguild prey was inferior to that of extraguild prey. For example, *Phytoseiulus persimilis* (Athias-Henriot), *Neoseiulus cucumeris* (Oudemans), *Amblyseius andersoni* (Chant) and *Neoseiulus fallacis* (Garman) have already been considered low nutrient food sources when used as intraguild prey. Although they allowed their intraguild predators to develop into adulthood, they did not enable them to reproduce [17,18]. However, during periods of natural prey scarcity, an intraguild prey such as *Amblyseius swirskii* (Athias-Henriot) is an equally nutritious food compared with extraguild prey, as has been observed in the case of *Frankliniella occidentalis* (Pergande) [19]. Obtaining nutritional benefits from intraguild prey can increase the survival of predators during spatiotemporal scarcity of extraguild prey. Evaluating the influences of intraguild prey on the fitness of an intraguild predator in a guild can improve our understanding of the interspecific interactions and improve the forecast of persistence potential of predator populations during prey scarcity.

Predatory thrips and mites, including *Scolothrips takahashii* (Priesner) (Thysanoptera: Thripidae) and *Neoseiulus barkeri* (Hughes) (Mesostigmata: Phytoseiidae), are natural enemies used for controlling populations of two-spotted spider mites, *Tetranychus urticae* (Koch) (Acari: Tetranychidae) [20,21,22]. *Scolothrips takahashii* suppressed *T. urticae* populations at low levels in various crops, such as citrus, pear, apple and bean in China [23,24]. The largest domestically produced indigenous predatory mite, *N. barkeri*, has been progressively released in orchards and greenhouses to control *T. urticae* populations before the spider mite damage becomes visible [25,26]. Both native enemies *S. takahashii* and released enemies *N. barkeri* frequently co-occur in the same ecosystem at low target prey densities, potentially leading to intraguild predation among the different life stages of the species in question. Previous studies have shown that intraguild interaction between *S. takahashii* and *N. barkeri* was bidirectional, with each species fed on the vulnerable stage of the other [27]. However, it remains unknown whether this behavior of theirs can lead to a higher fitness of the intraguild predators, and consequently increase their persistence during periods of target prey scarcity.

In this study, we investigated the effects of *N. barkeri* eggs and first instar larvae of *S. takahashii*, which are the vulnerable stages of these intraguild prey, on the survival, juvenile development and female reproduction of their intraguild predator *S. takahashii* and *N. barkeri*, respectively. Furthermore, the preference of the intraguild predators for intraguild prey or *T. urticae* was also evaluated.

## 2. Materials and Methods

### 2.1. Rearing of Mites and Thrips

The two-spotted spider mite (*T. urticae*) was reared on cowpea bean plants *Vigna unguiculata* (L.). *Neoseiulus barkeri* were reared on a black polyethylene plastic sheet placed upside down on a water-soaked sponge inside a plastic container (15 × 20 × 10 cm), which was sealed by a lid with a fine mesh (150 mm mesh opening) as window in the middle to allow for ventilation. Sufficient numbers of mixed stages of *T. urticae* were brushed into the predatory mite cultures as food every other day. Colonies of *S. takahashii* were maintained on bean plants *V. unguiculata* infested by *T. urticae*. Rearing units were maintained at 28 ± 1 °C, 80 ± 5% R.H. and 16L: 8D photoperiod.

### 2.2. Experimental Design and Procedures

The experiments were carried out in closed arena, which were modified from a previous study [28]. The experimental set-up consisted of two clear acrylic plates (4 × 3 × 0.3 cm) that overlapped each other and had a round hole (Ø = 2.5 cm) in the center. A fresh bean leaf was placed between these two acrylic plates, which were then covered with a transparent acrylic sheet (3 × 3 × 0.1 cm) on the upper side. Those plates were held together with 19 mm foldback clips at two opposite sides. The experimental cells were placed in a plastic tray (30 × 25 × 3 cm) with a double-distilled water-soaked sponge (25 × 20 × 1 cm) to keep the leaves fresh. Experimental arenas were maintained at 28 ± 1 °C, 80 ± 5% R.H. and 16L: 8D photoperiod.

According to our previous study, eggs of *N. barkeri* and first instar larvae of *S. takahashii* were the most vulnerable respective stages and were the preferred intraguild prey in guilds [27]. *Neoseiulus barkeri* preferred the protonymphs of *T. urticae* to other developmental stages when faced with interspecific predation risk from predatory thrips. Additionally, *S. takahashii* preyed on all stages of *T. urticae*, with a higher predation rate on protonymphs of *T. urticae* when exposed to interspecific predation risk from *N. barkeri* (unpublished data). Thus, the protonymphs of *T. urticae* served as extraguild prey. For the development experiment, we used protonymphs of *N. barkeri* and first instar larvae of *S. takahashii* as intraguild predators. Adult females of both predators were used as intraguild predators for the oviposition experiment and choice test.

#### 2.2.1. Survival and Development of Immature Intraguild Predators

Newly laid eggs of *N. barkeri* were transferred to each experimental cell (as described). As soon as the obligatory feeding stage (protonymphs stage) was reached, they were fed by randomly assigning them one of the three following treatments: sufficient first instar larvae of *S. takahashii* as intraguild prey; *T. urticae* protonymphs as extraguild prey; or no food. Similarly, newly emerged first instar larvae of *S. takahashii* in each cell were offered with sufficient *N. barkeri* eggs as intraguild prey, *T. urticae* protonymphs as extraguild prey, or no food. These experimental cells were monitored twice a day to record the survival and development of the intraguild predators in each cell. To comprehensively evaluate the survival and development time of the intraguild predator’s immature stage, we also recorded the duration of the unfed stage (including *N. barkeri* eggs and larvae, as well as *S. takahashii* prepupa and pupa) in each treatment. The observation was terminated when the intraguild predators *N. barkeri* and *S. takahashii* reached adulthood or died. Every day the experimental cells were renewed and the prey replenished. For both intraguild predator species, 15 replicates were set up for each treatment.

#### 2.2.2. Survival and Oviposition of Adult Female Intraguild Predators

One female (9–11 days old since egg hatching) of each predator species was transferred into a detached bean leaf disc with ample prey of mixed stages of *T. urticae*, and one conspecific male was introduced into each leaf disc for mating. After 24 h (until mating was observed), each female was transferred into a fresh leaf disc with no food to standardize their level of hunger. Only females that deposited at least one egg during the starvation period were used as intraguild predators. Subsequently, starved intraguild predators were introduced to experimental cells, and the intraguild prey or natural prey were separately added into each arena. The survival and oviposition of each female was recorded daily for up to 10 days. The experimental cell was replaced daily with sufficient prey as before for each tested individual. Oviposition of females was also measured when they were offered no prey. Each treatment was replicated 15 times for each prey.

#### 2.2.3. Prey Preference of Intraguild Predators

Gravid females of *N. barkeri* randomly selected from the rearing units described above were subjected to starvation for 24 h. Subsequently, gravid females of *N. barkeri* were singly introduced into an experimental cell supplied with intraguild prey and extraguild prey at a ratio of 1:1 (ten first instar larvae of *S. takahashii* and ten active stage protonymphs of *T. urticae*). The same procedure was employed for *S. takahashii* females, except that prey provision was doubled (20 eggs of *N. barkeri* and 20 active stage protonymphs of *T. urticae*). The experimental cell was observed every 10 min for 6 h or until the intraguild prey or extraguild prey was attacked by the intraguild predator. Prey preference was determined based on the observation of the first three attacks [29]. After each attack, the attacked prey was replaced by another individual to keep the prey density and relative abundance constant. Attack frequency was calculated as the proportion of a specific prey (intraguild prey or extraguild prey) being attacked by the intraguild predator. Fourteen replicates were carried out for each intraguild predator.

### 2.3. Data Analysis

All analyses were performed using SPSS 21.0. To compare the cumulative survival rates of intraguild predators under different types of diet (no food, intraguild prey and extraguild prey), Kaplan–Meier survival analysis was performed using Log-rank tests. T-test for independent samples was used to compare the two types of prey (intraguild prey and extraguild prey) on the developmental parameters of the intraguild predators: including *N. barkeri* eggs, total duration of development from egg to adult, and—for *S. takahashii*—first instar larvae, second instar larvae and total duration of development from first instar larvae to adult. A Mann–Whitney U-test was performed to compare the development time of *N. barkeri* larvae, protonymphs and deutonymphs, and *S. takahashii* prepupa and pupa when provided with two types of prey, because the data could not be normalized. The oviposition rates of adult females of intraguild predators provided with either extraguild prey or intraguild prey were also compared using the T-test for independent samples. Data from the first day were omitted from the calculations of oviposition capacity to reduce the influence of previous feeding history. Results of the choice experiment were analyzed through the chi-square test.

## 3. Results

### 3.1. Survival and Development of Immature Intraguild Predators

**Figure 1:**
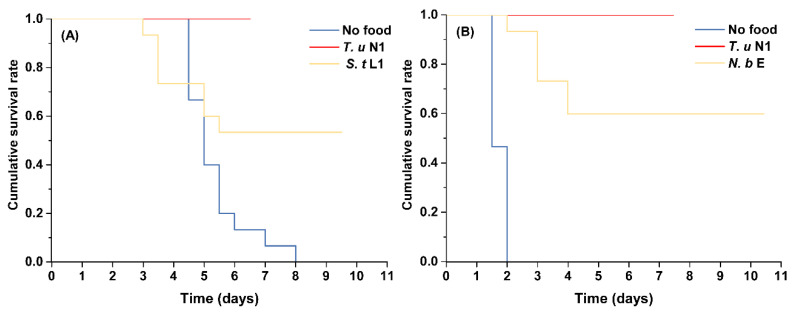
Cumulative survival rates of immature *Neoseiulus barkeri* (**A**) and *Scolothrips takahashii* (**B**) fed with intraguild prey (*S. takahashii* first instar larva served as intraguild prey for *N. barkeri, N. barkeri* egg served as intraguild prey for *S. takahashii*), extraguild prey (*Tetranycus urticae* protonymph), or no food. *T. u*: *T. urticae*; *S. t*: *S. takahashii*; *N. b*: *N. barkeri*; N1: protonymph; L1: first instar larva; E: egg. The cumulative survival rates of intraguild predators under different types of diet were compared with Kaplan–Meier survival analysis using Log-rank tests (*p* < 0.05).

The survival rate of both intraguild predators when fed on prey was significantly higher than when they were deprived of food (*N. barkeri* as intraguild predator overall test, as shown on Figure 1A: *χ*^2^ = 14.979, *df* = 2, *p* = 0.001; *T. urticae* protonymph as extraguild prey vs. no food: *χ*^2^ = 11.911, *p* = 0.001; *S. takahashii* first instar larva as intraguild prey vs. no food: *χ*^2^ = 4.851, *p* = 0.028; *S. takahashii* as intraguild predator overall test, as shown on Figure 1B: *χ*^2^ = 49.999, *df* = 2, *p* < 0.001; *T. urticae* protonymph as extraguild prey vs. no food: *χ*^2^ = 29.578, *p* < 0.001; *N. barkeri* egg as intraguild prey vs. no food: *χ*^2^ = 26.729, *p* < 0.001). Moreover, the intraguild predators that were provided with intraguild prey had a lower survival rate than the intraguild predators fed on extraguild prey (*N. barkeri* as an intraguild predator, Figure 1A: *T. urticae* protonymph as extraguild prey vs. *S. takahashii* first instar larva as intraguild prey: *χ*^2^ = 6.259, *p* = 0.012; *S. takahashii* as an intraguild predator, Figure 1B: *T. urticae* protonymph as extraguild prey vs. *N. barkeri* egg as intraguild prey: *χ*^2^ = 7.266, *p* = 0.007).

There were significant differences in the durations of protonymph (*U* = 7.500, *p* < 0.001), deutonymph (*U* = 9.500, *p* < 0.001) and the overall development time (*t* = −5.085, *p* < 0.001) among *N. barkeri* resulting from the different food treatments (Table 1). Similarly, significant differences were observed in the development time of *S. takahashii*, including first instar larva (*t* = −7.130, *p* < 0.001), second instar larva (*t* = −2.381, *p* = 0.041) and the overall development time (*t* = −3.708, *p* = 0.004, Table 2).

### 3.2. Survival and Oviposition of Adult Female Intraguild Predators

**Figure 2:**
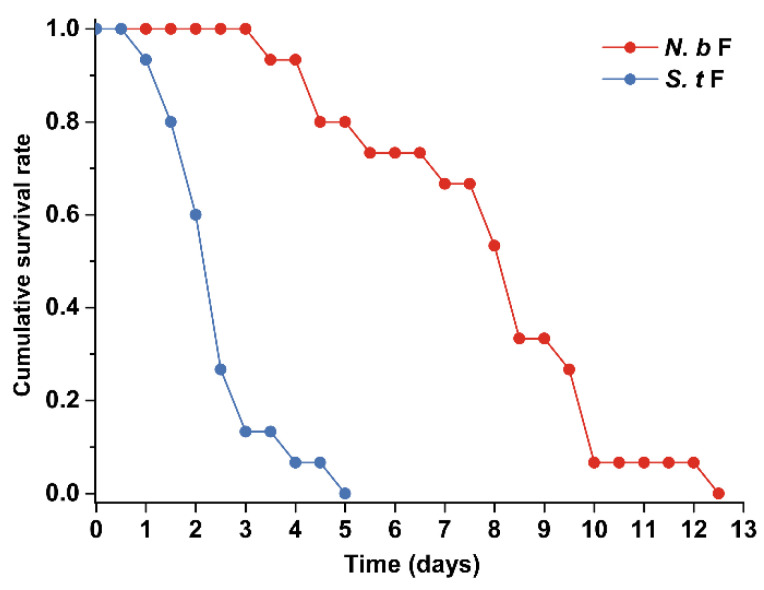
Cumulative survival rates of female *Neoseiulus barkeri* and *Scolothrips takahashii* subject to no food. *N. b*: *N. barkeri*; *S. t*: *S. takahashii*; F: female.

**Figure 3:**
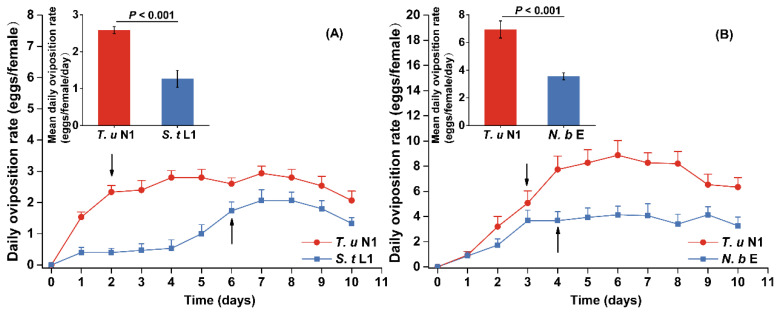
The oviposition (mean ± SE) of females of *Neoseiulus barkeri* (**A**) and *Scolothrips takahashii* (**B**) when offered intraguild prey (*S. takahashii* first instar larva served as intraguild prey for *N. barkeri* female, *N. barkeri* egg served as intraguild prey for *S. takahashii* female) and extraguild prey (*Tetranychus urticae* protonymph). *T. u*: *T. urticae*; *S. t*: *S. takahashii*; *N. b*: *N. barkeri*; N1: protonymph; L1: first instar larva; E: egg. T-test for independent samples was used to measure the oviposition per female per day in different diets (*p* < 0.05). The arrow points to the time at which all predators resumed the ability to lay eggs.

When prey was unavailable, no egg was laid by either intraguild predators. All *S. takahashii* females died within 5 days, while *N. barkeri* females died within 12.5 days (Figure 2). By contrast, all female intraguild predators provided with prey survived throughout the experiment and laid eggs (Figure 3). In addition, the time for the predators to restore their egg laying ability differed significantly according to their prey types. All *N. barkeri* and *S. takahashii* females started to lay eggs on the second or third day, respectively, after being provided with natural prey *T. urticae* protonymph; but resumed egg production on the 6th and 4th day, respectively, when provided with intraguild prey.

Oviposition rates of both intraguild predator species were higher when they were provided with extraguild prey (Figure 3). During the experiment, *Neoseiulus barkeri* laid more eggs when given *T. urticae* protonymph than when given first instar larvae of thrips (*t* = 5.324, *p* < 0.001). Similarly, *S. takahashii* had a higher oviposition rate when fed on extraguild prey than on intraguild prey (*t* = 5.077, *p* < 0.001).

### 3.3. Prey Preference of Intraguild Predators

**Figure 4:**
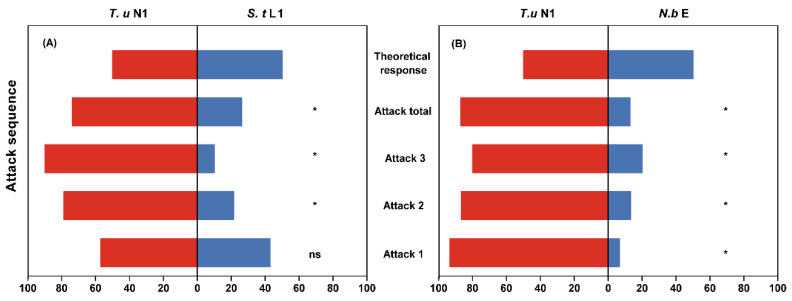
Preference experiments. Attack frequencies of *Neoseiulus barkeri* (**A**) and *Scolothrips takahashii* (**B**) on intraguild prey (*S. takahashii* first instar larva served as intraguild prey for *N. barkeri* female, *N. barkeri* egg served as intraguild prey for *S. takahashii* female) and extraguild prey (*Tetranychus urticae* protonymph). Attack 1, 2, 3 and total were calculated as the proportion of intraguild predator first attack, second attack, third attack and the sum of these three attacks on a specific prey. Theoretical response represents no prey preference by the intraguild predator for each specific prey (theoretical index of 50%). *T. u*: *T. urticae*; *S. t*: *S. takahashii*; *N. b*: *N. barkeri*; N1: protonymph; L1: first instar larva; E: egg. Red areas show predation on the *T. urticae*, blue areas show predation on intraguild prey. Asterisks indicate a significant prey preference (*χ*^2^, *p* < 0.05).

*Neoseiulus barkeri* exhibited a preference for extraguild prey over intraguild prey (the second attack frequency: *χ*^2^ = 4.571, *p* = 0.033; the third attack frequency: *χ*^2^ = 6.400, *p* = 0.011; and the total attack frequency: *χ*^2^ = 8.526 *p* = 0.004, Figure 4A). However, there was no significant difference in the first attack frequency of *N. barkeri* on *T. urticae* protonymph compared to thrips first instar larvae (*χ*^2^ = 0.286 *p* = 0.593). The attack frequency of *S. takahashii* was significantly higher on extraguild prey *T. urticae* than on intraguild prey *N. barkeri* egg (the first attack frequency: *χ*^2^ = 11.267, *p* = 0.001; the second attack frequency: *χ*^2^ = 8.067, *p* = 0.005; the third attack frequency: *χ*^2^ = 5.400, *p* = 0.020; and the total attack frequency: *χ*^2^ = 24.200, *p* < 0.001, Figure 4B).

## 4. Discussion

This study showed that most individuals from both the *N. barkeri* and *S. takahashii* species successfully developed and reproduced when fed on their extraguild and intraguild prey. However, both *N. barkeri* and *S. takahashii* showed higher survival rates, faster development time, and higher oviposition rates when fed on extraguild prey than on intraguild prey. The choice test revealed that both predator species preferred the spider mites *T. urticae*. These results indicate that intraguild prey could serve as an alternative food source for *N. barkeri* and *S. takahashii* when target prey becomes scarce, thereby prolonging survival time and ensuring reproduction of the predators. These findings support the theory that intraguild predation is an adaptative strategy for the predators under conditions of prey scarcity [3].

The nutritional benefits of intraguild prey for the predators *N. barkeri* and *S. takahashii* allow juveniles to reach the adult stage and females to reproduce. In this study, the predator *N. barkeri* fed on first instar larvae of *S. takahashii* displayed a similar development time to those fed on *T. urticae* nymphs [30] and *Eotetranychus kankitus* (Ehara) of mixed stages [31]. In addition, *N. barkeri* was capable of maintaining oviposition (1.26 eggs per female per day) when fed on intraguild prey first instar larvae of *S. takahashii;* and their oviposition rate was similar to that when they were fed on other prey such as *Tyrophagus putrescentiae* (Schrank) [32], *Aleuroglyphus ovatus* (Troupeau) [33] and *Luffa cylindrical* (Roem) pollen [34]. Thus, intraguild prey can prolong the survival of *N. barkeri* when their food sources such as tetranychid and Acaridae prey or pollen are scarce. The present results confirm the finding of Momen [35], who reported that *N. barkeri* juveniles completed their development, and female produced 1.35 eggs per female per day when fed on the intraguild prey *Typhlodromus negevi* (Swirski and Amitai) eggs. Other phytoseiid species—including *Typhlodromus athiasae* (Porath and Swirski) [36] and *N. cucumeris* [18]—were also observed to complete their ontogenetic development and to sustain oviposition on intraguild prey. These intraguild prey were considered as alternative prey contributing to the predator’s persistence under prey scarcity. However, intraguild prey may be considered only as supplemental food in some cases. For example, a study by Schausberger and Croft [18] reported that immature phytoseiid species, *Neoseiulus longispinosus* (Evans) and *Galendromus occidentalis* (Nesbitt), failed to develop into adults on intraguild prey. 

Previous studies have reported that the development time of *S. takahashii* was shorter when they were fed on *T. urticae* (5.50 days) [37] and *Panonychus citri* (McGregor) (5.57 days) [38] compared to when provided with *N. barkeri* eggs (8.167 days). However, *S. takahashii* fed on *N. barkeri* eggs laid 3.56 eggs per female per day, which was slightly less than that when fed on *P. citri* (5.06 eggs per female per day, 28 °C) [38] and *T. urticae* (5.06 eggs per female per day, 25 °C) [37]. Currently, no information is available on the interaction of *S. takahashii* with other phytoseiid mites or predatory insects. However, *Scolothrips longicornis* (Priesner), which belongs to the same genus as *S. takahashii*, showed a daily oviposition rate of 0.08 and 0.13 when fed on eggs of phytoseiid mites *Typhlodromus bagdasarjani* (Wainstein & Arutunjan) and *Neoseiulus californicus* (McGregor), respectively [39], whereas *S. takahashii* laid 3.56 eggs per female per day when fed on *N. barkeri* eggs. As a result, *S. takahashii* demonstrated its capacity to sustain a population over a long term by consuming *N. barkeri* eggs when the target prey was scarce.

Overall, both intraguild predator species preferred extraguild prey, which may be due to its high quality. Both predators showed higher survival rates, shorter development times and higher oviposition rates on extraguild prey than on intraguild prey. However, *N. barkeri* did not show a preference between extraguild prey and intraguild prey in their first attack. This may be attributed to the fact that *S. takahashii* larva is probably a suitable prey for the *N. barkeri*. However, *S. takahashii* larvae exhibited defensive behavior such as jerking the end of the abdomen rapidly or producing excrement during the experiment [40], which could lead *N. barkeri* to avoid them and then alter their preferences for thrips larvae in the second attack. This defensive behavior has also been observed in other insects such as *F. occidentalis* and *Thrips tabaci* (Lindeman) [41,42]. This result indicates that, although the first instar larvae of *S. takahashii* may have high nutritional value for *N. barkeri,* the difficulty in catching thrips makes them a less suitable food source.

The present study revealed that intraguild prey could ensure the survival, development, and oviposition of predators *N. barkeri* and *S. takahashii*, increasing their persistence period with prey scarcity, although extraguild prey was of higher quality for both predators. However, evaluating persistence and coexistence of predators in the presence of all the species and in arenas differing in spatial structure in the field warrants using experiments based on population/community dynamics, since these would allow more realistic predation rates to be compared under scenarios of intraguild predation. In addition, a polytypic diet would allow for a balanced nutritional intake for the predators [43]. Thus, intraguild prey may not only enable intraguild predators to survive when other prey is scarce but also to improve the fitness of predators when there is overabundance of prey. Further experiments are required to investigate the fitness of intraguild predators when provided with a polytypic diet consisting of a mix of intraguild prey and extraguild prey to clarify the benefits of intraguild prey for intraguild predators in a system with one prey and multiple predators.

## Figures and Tables

**Table 1:** Development time in days (means ± SE) of *Neoseiulus barkeri* fed on intraguild prey *Scolothrips takahashii* first instar larva and extraguild prey *Tetranychus urticae* protonymph.

Predator	Prey	Development Time (Days)				
		Egg	Larva	Protonymph	Deutonymph	Total
*N. b*	*T. u* N1	1.600 ± 0.100	0.700 ± 0.065	1.400 ± 0.072	1.200 ± 0.118	4.900 ± 0.240
	*S. t* L1	1.750 ± 0.095	0.625 ± 0.082	2.375 ± 0.227 *	2.375 ± 0.324 *	7.125 ± 0.3988 *
*t*		−0.974	-	-	-	−5.085
*U*		-	51.000	7.500	9.500	-
Sig.		0.341	0.482	<0.001	<0.001	<0.001

Note: *N. b*: *Neoseiulus barkeri*; *T. u*: *Tetranychus urticae*; *S. t*: *Scolothrips takahashii*; N1: protonymph; L1: first instar larva. Asterisks indicate differences in development time of *N. barkeri* fed on different types of prey (T-tests for independent samples was used to analyze development times of the *N. barkeri* egg and the total duration of development from egg to adult, and a Mann–Whitney U-test was performed for *N. barkeri* larva, protonymph and deutonymph development time, *p* < 0.05).

**Table 2:** Development time in days (means ± SE) of *Scolothrips takahashii* fed on intraguild prey *Neoseiulus barkeri* egg and extraguild prey *Tetranychus urticae* protonymph.

Predator	Prey	Development Time (Days)				
		First instar larva	Second instar larva	Prepupa	Pupa	Total
*S. t*	*T. u* N1	1.767 ± 0.067	1.867 ± 0.133	0.867 ± 0.059	1.633 ± 0.059	6.133 ± 0. 192
	*N. b* E	2.778 ± 0.147 *	3.111 ± 0.505 *	0.667 ± 0.083	1.611 ± 0.073	8.167 ± 0.514 *
*t*		−7.130	−2.381	-	-	−3.708
*U*		-	-	40.500	64.500	-
Sig.		<0.001	0.041	0.060	0.812	0.004

Note: *S. t*: *Scolothrips takahashii*; *T. u*: *Tetranychus urticae*; *N. b*: *Neoseiulus barkeri*; N1: protonymph; E: egg. Asterisks indicate differences in development time of *S. takahashii* fed on different types of prey (T-tests for independent samples were used to compared development times of *S. takahashii* first instar larva, second instar larva and the total duration of development from first instar larvae to adult, and a Mann–Whitney U-test was performed for *S. takahashii* prepupa and pupa development time, *p* < 0.05).

## Data Availability

The data presented in this study are available on request from the corresponding author.
